# Annual N95 respirator fit-testing: an unnecessary burden on healthcare

**DOI:** 10.1017/ice.2023.187

**Published:** 2024-02

**Authors:** Thomas C. S. Martin, Genevieve Curtin, Natasha K. Martin, Francesca J. Torriani

**Affiliations:** 1 University of California San Diego, San Diego, California, USA; 2 Veterans’ Affairs San Diego, San Diego, California, USA

## Abstract

US regulations mandate annual N95 mask fit testing for healthcare workers, but the optimal testing interval is unknown. In our study using data from 12,565 healthcare workers, the probability of survival free from fit-test failure after 3 years was 99.4%, suggesting that less frequent fit testing every 3 years would be safe.

Healthcare transmission of airborne respiratory diseases, in particular *Mycobacterium tuberculosis* (TB), has been described in a variety of healthcare settings.^1,2^ The control of airborne respiratory infection transmission in healthcare settings is achieved through a combination of administrative measures (eg, moving persons with the possibility of infection to single-patient rooms), engineering measures (eg, negative-pressure rooms, hospital ventilation and high-efficiency particulate air [HEPA] filters), and respiratory protection devices (eg, N95 respirators).

The effectiveness of N95 respirators in the prevention of TB and other airborne infections has not been well established but remains the standard of practice in most healthcare settings.^1,3,4^ In a study that evaluated the hospital transmission of TB in the late 1980s and 1990s during implementation of control measures, administrative and engineering measures most likely led to the substantial decrease in transmission, rather than the use of respiratory protection devices.^1^ In addition, comparisons between N95 respirators and surgical masks in the transmission of laboratory-confirmed respiratory infection, influenza-like illness or workplace absenteeism have not demonstrated a significant difference.^3,4^ However, more recent work has indicated the utility of N95 respirators in the prevention of SARS-CoV-2 transmission in a combination of healthcare and nonhealthcare settings.^5^

Fit-testing of respirators with at least annual retesting is mandated prior to the usage of N95 respirators for all healthcare workers with a risk of airborne pathogen exposure in the United States.^6^ The laboratory performance of respirators is improved with fit-testing strategies; however, data are limited regarding the rate of subsequent test failure, which limits decision making regarding the frequency of retesting.^7–9^ A study of 229 participants revealed a significant increase in risk of fit-test failure over time, with ∼25% experiencing fit-test failure after 3 years.^9^ We evaluated the probability of failing respirator fit test over time among a population of healthcare workers in Southern California.

## Methods

### Population and setting

In this study, we included all adult (>18 years old) healthcare employees required to undergo annual respiratory fit testing according to California Occupational Safety and Health Administration (Cal-OSHA) from University of California San Diego Health (UCSDH) who had 2 or more fit test results.

### Data abstraction and analysis

**Figure 1.**
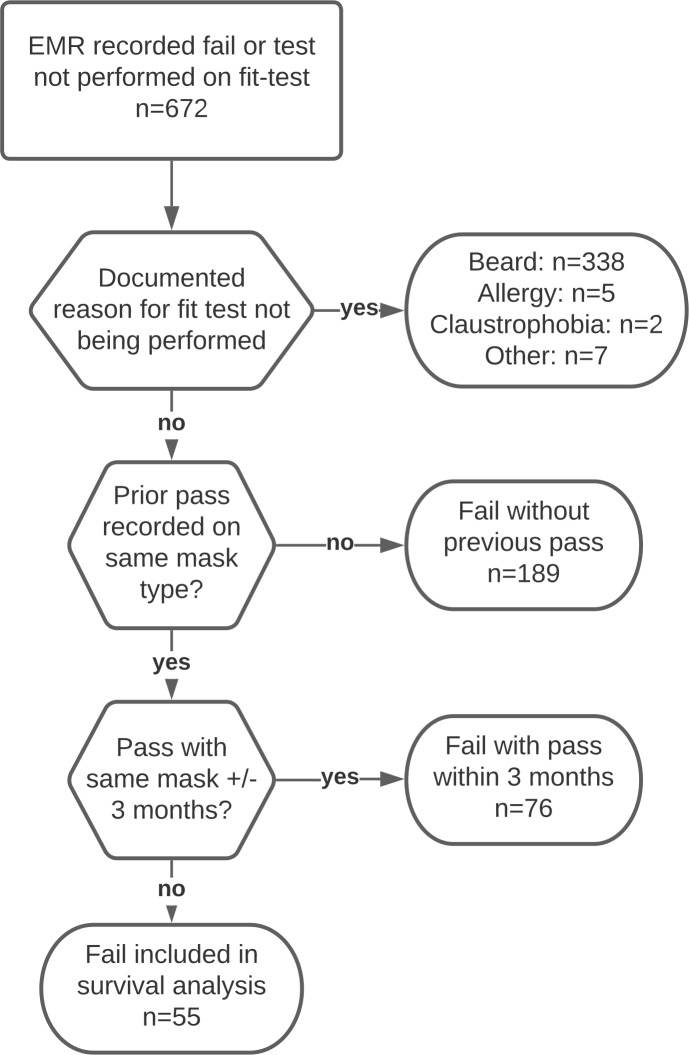
Flow chart indicating how fit-test failures or fit-test not performed were ultimately adjudicated using electronic medical record, paper documentation, and powered air-puryfying respirator (PAPR) referral forms.

Fit-test outcomes were abstracted from electronic medical records for all participants. Where needed to clarify outcomes, paper records and referrals for a powered air-puryfying respirator (PAPR) were reviewed. All fit-tests were performed on 1 of 5 respirators (3M1870, 3M1870+ Aura, 3M1860, 3M1860+, Gerson2130) using OSHA-approved qualitative fit-testing protocols using saccharin and/or denatomium benzoate solutions (Bitrex, Edinburgh, Scotland).^6^ Fit-test outcomes were categorized using the strategy outlined in Figure 1 to identify fit-test failures versus other reasons for noncompletion of a fit-test, such as the presence of facial hair. For inclusion in the survival analysis, individuals had to have a recorded pass on a mask with at least 1 subsequent test on the same mask. If a fit-test pass and a fit-test fail were recorded on the same mask within 3 months, we adjudicated the fail as a noncompletion of a fit test. For each mask type, the median follow-up duration was calculated. Kaplan-Meier survival analysis was used to estimate the probability of survival free from fit-test failure at 3 and 5 years after a pass on the same mask.

### Ethics

A waiver of consent for the study was granted by the institutional review board prior to data abstraction (project no. 190680X).

## Results

Overall, 15,757 persons with at least 1 fit-test result were identified. Among these persons, 49,290 fit-test results were recorded: 48,615 (98.6%) were passes, 323 were fails (0.7%), and 352 (0.7%) were not completed. Also, 3 failures were reclassified as passes after review of electronic medical record documentation. From 352 fit tests that were not completed, 338 were due to the presence of a beard, 5 were due to history of allergy, 2 were due to claustrophobia, and 7 were due to reasons unrelated to the fit of the mask. From 320 recorded fit-test fails, 189 had no prior pass on the same respirator and 76 had a pass recorded on the same respirator within 3 months (Fig. 1).

We identified 55 instances of fit-test failure after a prior pass on the same mask occurring >3 months previously. These 55 fails were incorporated into the survival analysis. Notably, among these fit-test fails included in the survival analysis, 19 had a subsequent recorded pass with the same respirator >3 months after the recorded failure.

**Figure 2.**
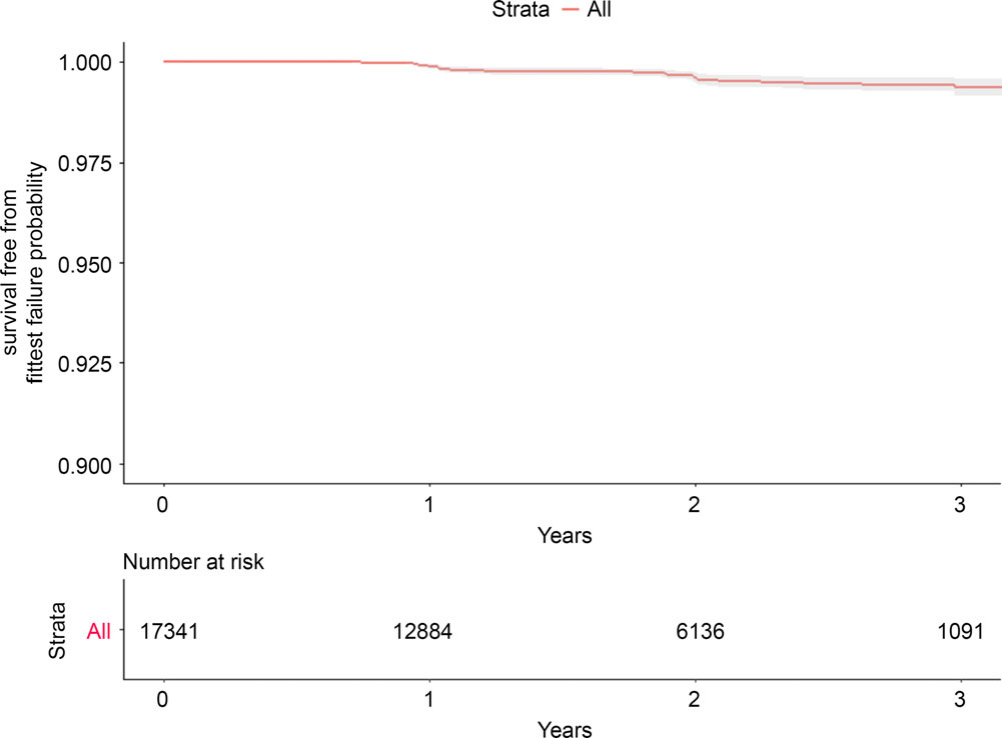
Kaplan-Meier survival analysis showing probability of survival free from fit-test failure including all mask types.

The survival analysis included 12,065 individuals representing 31,270 person mask-years of follow-up. The median follow-up period across all respirator types was 1.9 years (interquartile range, 1.0–2.7). At 3 years, 54 events were recorded, with a probability of survival free from fit-test failure of 99.4% (95% confidence interval [CI], 99.2%–99.6%) (Fig. 2). Although fewer individuals were followed, the 5-year probability of survival free from fit-test failure was estimated at 99.4% (95% CI, 99.2%–99.6%).

## Discussion

After fit-testing for a N95 respirator, the probability of fit-test failure on the same respirator within 3 years is likely to be less than 0.5%. From the 55 failures recorded, a significant proportion (35%) went on to record a pass with the same mask on subsequent testing. Our results call into question the rationale behind the mandated annual fit-testing for N95 respirators which was based on one small study of 229 participants.^9^

The cost of annual testing is likely to be very substantial requiring equipment, full-time staff, and physical infrastructure to perform the testing. We estimated that the total annual cost to healthcare in the United States for fit testing is in the range of US$ 200–400 million for infrastructure, salaries, and equipment.^10^ In addition to the costs in this estimate, the time commitment from individuals coming to be tested is likely to be at least 30–60 minutes each year, which would contribute further to overall program cost. The potential saving from moving to screening every 3 years could therefore be very substantial.

Our study had several limitations. The study was retrospective; we relied on historical documentation for the assessment of fit-test outcomes. In many cases, there was no documentation of the reason for fit-test failure, which likely led to an overestimation of the true failure rate. This finding is supported by the finding that many ‘fails’ subsequently passed on the same respirator type. Due to changes in respirator usage over time at our institution, the available follow-up time on each respirator type used limits our ability to draw conclusions about a testing strategy beyond 3 years. Further research would be needed to assess the safety of testing less frequently than every 3 years. Finally, this study was not powered to detect whether more or less frequent fit testing has a meaningful impact on the rate of acquisition of airborne infections.

In summary, we have demonstrated that N95 respirator fit-test failure after a previous pass within 3 years is rare. Our results support a change in policy to reduce the fit-testing interval mandate from annual to every 3 years.
